# De novo and inherited variants in coding and regulatory regions in genetic cardiomyopathies

**DOI:** 10.1186/s40246-022-00420-0

**Published:** 2022-11-10

**Authors:** Nirmal Vadgama, Mohamed Ameen, Laksshman Sundaram, Sadhana Gaddam, Genomics England Research Consortium, Casey Gifford, Jamal Nasir, Ioannis Karakikes

**Affiliations:** 1 Department of Cardiothoracic Surgery and Cardiovascular Institute, Stanford University, 240 Pasteur Drive, Palo Alto, CA 943054 USA; 2 Department of Pediatrics, Division of Cardiology, Stanford School of Medicine, Lucile Packard Children’s Hospital, Palo Alto, CA USA; 3 Department of Genetics, Stanford School of Medicine, Lucile Packard Children’s Hospital, Palo Alto, CA USA; 4 BASE Initiative, Betty Irene Moore Children’s Heart Center, Lucile Packard Children’s Hospital, Palo Alto, CA USA; 5 Department of Cancer Biology, Stanford University, Stanford, CA USA; 6 Department of Computer Science, Stanford University, Stanford, CA USA; 7 Program in Epithelial Biology and Department of Dermatology, Stanford University School of Medicine, Stanford, CA USA; 8 Genomics England, London, UK; 9 Division of Life Sciences, University of Northampton, Waterside Campus, University Drive, Northampton, NN1 5PH UK

**Keywords:** Cardiomyopathy, Single-cell, Noncoding, De novo, Regulome, Oligogenic

## Abstract

**Background:**

Cardiomyopathies are a leading cause of progressive heart failure and sudden cardiac death; however, their genetic aetiology remains poorly understood. We hypothesised that variants in noncoding regulatory regions and oligogenic inheritance mechanisms may help close the diagnostic gap.

**Methods:**

We first analysed whole-genome sequencing data of 143 parent–offspring trios from Genomics England 100,000 Genomes Project. We used gene panel testing and a phenotype-based, variant prioritisation framework called Exomiser to identify candidate genes in trios. To assess the contribution of noncoding DNVs to cardiomyopathies, we intersected DNVs with open chromatin sequences from single-cell ATAC-seq data of cardiomyocytes. We also performed a case–control analysis in an exome-negative cohort, including 843 probands and 19,467 controls, to assess the association between noncoding variants in known cardiomyopathy genes and disease.

**Results:**

In the trio analysis, a definite or probable genetic diagnosis was identified in 21 probands according to the American College of Medical Genetics guidelines. We identified novel DNVs in diagnostic-grade genes (*RYR2, TNNT2, PTPN11, MYH7, LZR1, NKX2-5*), and five cases harbouring a combination of prioritised variants, suggesting that oligogenic inheritance and genetic modifiers contribute to cardiomyopathies. Phenotype-based ranking of candidate genes identified in noncoding DNV analysis revealed *JPH2* as the top candidate. Moreover, a case–control analysis revealed an enrichment of rare noncoding variants in regulatory elements of cardiomyopathy genes (p = .035, OR = 1.43, 95% Cl = 1.095–1.767) versus controls. Of the 25 variants associated with disease  (*p*< 0.5), 23 are novel and nine are predicted to disrupt transcription factor binding motifs.

**Conclusion:**

Our results highlight complex genetic mechanisms in cardiomyopathies and reveal novel genes for future investigations.

**Supplementary Information:**

The online version contains supplementary material available at 10.1186/s40246-022-00420-0.

## Background

The cardiomyopathies, herein divided into dilated cardiomyopathy (DCM), hypertrophic cardiomyopathy (HCM), arrhythmogenic right ventricular cardiomyopathy (ARVC) and left ventricular non-compaction cardiomyopathy (LVNC), are leading causes of heart failure [1].

Although there is considerable overlap between different cardiomyopathy subtypes, each has a signature genetic aetiology. HCM and ARVC are largely explained by alterations in sarcomere or desmosome proteins, respectively. Around half of HCM cases are caused by mutations in the genes *MYH7* and *MYBPC3* [2]. However, the genetic architecture of DCM is far more complex. To date, more than 250 genes have been implicated in DCM causation or risk, including genes encoding for cytoskeletal, sarcolemmal, mitochondrial, calcium cycling, costameric and sarcomeric proteins [3].

Although the aetiological basis of cardiomyopathy is incomplete, recent genetic studies suggest that a large proportion of cases may be explained by alterations in the noncoding genome [4].

Data from the ENCODE project suggest that biochemical functionality could be assigned to 80 per cent of the human genome, affecting regulatory and tissue-specific expression patterns [5]. Furthermore, genome-wide association studies (GWAS) show that over 90% of disease-associated SNPs are in noncoding regions of the genome, including regulatory regions, such as promoters and enhancers [6]. Thus, a key priority in the cardiomyopathy gene discovery pipeline is the identification of regulatory elements controlling genes associated with these disorders.

High-throughput epigenomic profiling methods such as ATAC-seq and ChIP-seq have enabled profiling of chromatin accessibility across samples in a tissue-wide manner, providing the opportunity to identify millions of context-specific regulatory elements. However, these bulk measurements of chromatin accessibility limit the precise understanding of how tissue heterogeneity and multiple cell types in the population contribute to overall disease aetiology [7]. Recent advances in single-cell ‘omics technologies have enabled an unbiased identification of cell-type populations and regulatory elements in a heterogeneous biological sample. By mapping the chromatin-regulatory landscape at a single-cell resolution, studies have demonstrated the potential to link regulatory elements to their target genes, and map regulatory dynamics during complex cellular differentiation processes [7–9].

We first performed parent–offspring trio analysis to assess the impact of rare inherited recessive and dominant variants, and of DNVs on cardiomyopathy. We hypothesised that variants in regulatory regions that are specifically active in the adult heart could provide an aetiological basis for cases with unexplained genetics. We further supported this hypothesis through systematic examination of noncoding regulatory elements of known disease-risk genes in a mutation-negative cardiomyopathy cohort. We identified noncoding variants predicted to disrupt *cis*-regulatory elements involved in cardiac gene regulation.

By combining inherited and DNV analysis of a clinically well-defined cohort, this study provides novel insights into the complex genetics of cardiomyopathy subtypes, which may lead to improved diagnosis and therapies.

## Materials and methods

### Study participants

All participants were recruited to the 100,000 Genomes Project (100KGP) (protocol version 7, 2020), with written informed consent. The full protocol is available online at https://doi.org/10.6084/m9.figshare.4530893.v7. Probands of parent–offspring trios (n = 143) and singleton offspring (n = 843) were diagnosed with either ARVC, LVNC, HCM, DCM or DCM and conduction defects. They were included in the study if they had a clear diagnosis under 40 years of age. Patients were excluded if they had an unclear diagnosis or a history suggestive of a non-genetic cause. The control cohort included 19,467 age-, sex-, and ethnicity-matched participants with no known heart disease.

The study adheres to the principles set out in the Declaration of Helsinki. Patients and relatives gave written informed consent for genetic testing. Ethical approval for the 100KGP was granted by the East of England—Cambridge South Research Ethics Committee (REC Ref 14/EE/1112).

### Initial data processing and gene selection

Whole-genome sequencing (WGS) were processed by Illumina, and sequencing data were passed through 100KGP's bioinformatics pipeline for alignment, annotation, and variant calling. Variants were prioritised using prespecified virtual gene panels from PanelApp (https://www.genomicsengland.co.uk). To date, 208 genes are listed for the Cardiomyopathies—including childhood onset (v1.37) panel. Based on the Human Phenotype Ontology (HPO) terms entered for these patients, additional gene panels were applied where applicable, including: undiagnosed metabolic disorders (v1.95), mitochondrial disorders (v1.127), intellectual disability (v3.2), and RASopathies (v1.27). Inherited variants were restricted to panel genes, where the allelic state was required to match the curated mode of inheritance.

### Identification of candidate variants in coding regions

The variants were categorised according to gene annotation, population allele frequency, functional prediction, and clinical interpretation. The raw list of SNVs and indels was annotated using ANNOVAR [10].

Variants were classified based on their mutational characteristics, position in the genome, allele frequency, and functional role in cardiomyopathy. In silico prediction of pathogenicity was performed using CADD [11] and REVEL [12], and conservation of nucleotides was scored using GERP +  +  [13]. Population allele frequencies were obtained from 100KGP, 1000Genomes, and gnomAD [14].

Highest priority was given to protein truncating (frameshift, stop gain, stop loss, splice acceptor variant, or splice donor variant) or de novo (protein truncating, missense, or splice region) variants in a gene on the diagnostic-grade list in the virtual gene panel for cardiomyopathy or any additional gene panel relevant to the phenotype to the patient [15].

Inherited protein-altering variants, such as missense and splice region variants, in diagnostic-grade genes were also considered. Variants were retained if they had a minor allele frequency (MAF) < 0.001; the allelic state matches the known mode of inheritance for the gene and disorder and segregates with disease (where applicable). Rare variants were ranked according to a REVEL score above the default threshold of 0.5, a CADD score greater than 20, and GERP +  + score greater than 2. Variants with clinical significance as benign or likely benign according to the ClinVar dataset were removed.

In parallel, we used Exomiser (v12.1.0) [16], a phenotype-driven variant prioritisation framework. Exomiser uses computational filters for variant frequency and predicted pathogenicity, protein interaction networks, patient phenotypes, cross-species phenotype comparisons, and pedigree information. A logistic regression model is used to combine the phenotype and variant scores to produce an overall Exomiser score. We considered the top three ranked variants that matched with our candidate-gene discovery analysis.

### De novo variant calling and filtering

DNVs were identified by 100KGP's bioinformatics pipeline. Briefly, variants from WGS data were called using Platypus, and filtered for absence of the mutation in both parents, read depth (> 20), allele balance (> 0.3 and 0.7), and no overlap with segmental duplications, simple repeat regions, and patch regions.

To analyse noncoding DNVs, we obtained single-cell ATAC and RNA data of human adult ventricle [14]. DNVs from 143 trios were intersected with the single-cell ATAC-seq peak sets using default parameters of bedtools v2.24.0. Peak sets were tested for an enrichment of DNVs in offspring as compared to a background peak set which contained peaks from all other cell types. We used a chi-squared test to compare the number of peaks with DNVs between the cardiomyocyte-specific peak set and the background peak set.

### HiChIP analysis

We used H3K27ac HiChIP to map active chromatin interactions genome wide on iPSC-derived cardiomyocytes (GSM3639703) [17]. HiChIP paired-end reads were aligned to GRCh38 genome using the HiC-Pro pipeline. Duplicate reads were removed and default HiC-pro settings were used to assign reads to MboI restriction fragments, filter for valid interactions, and generate binned interaction matrices. High-confidence contacts (FDR < 0.05) were called using the contact caller FitHiChIP with default settings at 10 kb resolution. These high-confidence contacts were used in visualisation.

### Prediction of target genes of enhancers

We used a combination of methods to predict enhancer–gene interactions and interpret the functions of noncoding DNVs.

Candidate enhancers were predicted using the recently developed activity-by-contact (ABC) model [18], which integrates H3K27ac ChIP-seq, HiChIP, and gene expression data with chromatin accessibility to predict enhancers and link them to their target genes. Using this method, we were able to identify sets of high-confidence putative enhancers for cardiomyocytes and their likely target genes.

In addition, publicly available Hi-C data of human left and right ventricle tissue (sample GSM1419085 and GSM2322554, respectively) [19, 20] were analysed in the 3D-genome Interaction Viewer (3DIV) and database (http://kobic.kr/3div/)20.

3DIV was run using distance-normalised interaction frequency ≥ 2 to define significant enhancer–promoter interactions. Topologically associating domains (TADs) were identified using TopDom [22] with a window size of 20. DNVs that were within enhancers predicted by both the ABC model and 3DIV were considered for downstream analysis. Finally, we used a machine learning approach called FATHMM-MKL [23] to predict the functional impact of noncoding SNVs. This tool integrates functional annotations from ENCODE with nucleotide-based sequence conservation measures and provides predictions as p values in the range 0 to 1. We used the default score > 0.5 to indicate putative deleterious variants.

### Promoters

Promoters were defined as 2 kb upstream or 1 kb downstream of transcription start sites (TSSs) and determined based on the basic gene annotation file of release 33 from GENCODE [24]. Further, to detect distal promoter-interacting loci we used promoter capture Hi-C data generated from three different human cell/tissue-types, including cardiomyocytes (GSM2297135, GSM2297136, GSM2297137, GSM2297138, GSM2297139), left ventricle (GSM2297192, GSM2297193, GSM2297194, GSM2297195, GSM2297196, GSM2297197, GSM2297198, GSM2297199, GSM2297200, GSM3067218, GSM3067219), and right ventricle (GSM2297289, GSM2297290, GSM2297291, GSM2297292, GSM2297293, GSM2297294, GSM2297295, GSM2297296, GSM2297297) [21].

### Network analysis

For noncoding DNVs, a functional enrichment analysis of the candidate genes was performed using the VarElect [25]. This tool uses the deep LifeMap Knowledgebase to infer the “direct” or “indirect” association of biological function between genes and the queried phenotype—i.e. “cardiomyopathy”. A direct association is determined if studies indicate that the gene in question directly affects disease development. An indirect association is based on factors such as shared pathways, protein–protein interaction networks, and mutual publications.

### Case-control analysis

Independent to the trio analysis, we analysed 843 probands and 19,467 unrelated controls to identify high-risk noncoding variants in regulatory elements of 12 cardiomyopathy genes with definitive (*BAG3, DES, FLNC, LMNA, MYH7, PLN, RBM20, SCN5A, TNNC1, TNNT2, TTN*) or strong (*DSP*) evidence [26]. We focused on regulatory elements of diagnostic genes rather than the entire genome to avoid false-positive results related to genes with an unclear association with the disease. Regulatory elements for each gene were determined using the ABC model (described above).

The functional impact of rare regulatory variants was assessed based on several tools, including RegulomeDB (https://regulomedb.org/), FunMotif (http://bioinf.icm.uu.se:3838/funmotifs/), and FATHMM-MKL. We mapped SNVs to these active regulatory regions of cardiomyopathy genes and defined them as high-risk if they were rare (MAF < 0.0001 in 100KGP and gnomAD population controls), predicted to alter transcription factor (TF) binding, and were enriched in cases versus controls (*p* < 0.05).

### Statistical analysis

To compare variant burden between cases and unrelated controls for high-risk regulatory variants of cardiomyopathy genes, variant calls were required to have an MAF of ≤ 0.0001 in 100KGP controls and gnomAD. Controls were proportionally matched for age, sex and ethnicity. *χ*^2^ test, odds ratios (OR), and 95% confidence intervals (95% CIs) were calculated for regulatory regions of all genes by comparing the burden of rare variants.

To evaluate the association between individual noncoding variants and the risk of cardiomyopathy, we performed a Fisher’s exact test as expected value were < 5. Statistical significance was considered at the 5% level (two-tailed). Statistical analyses were undertaken using R 4.2.0 and RStudio 2022.02.2.

## Results

### Demographics and phenotype data of probands

**Fig. 1:**
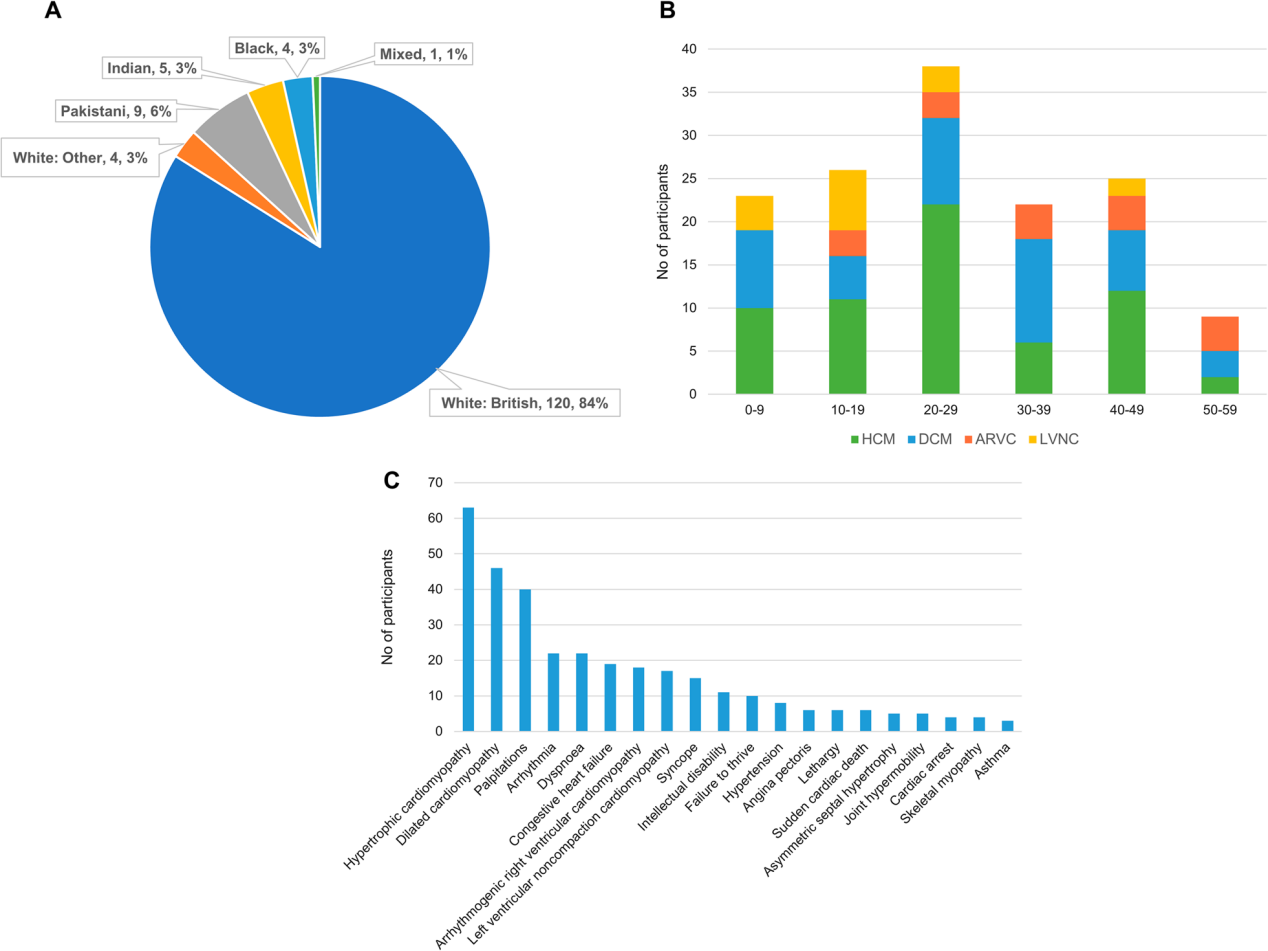
Proband demographics and phenotype in trio analysis. **A** Ethnicities recorded of participants. **B**, Age distribution of participants at present time and type of diagnosis made. **B** Top 20 Human Phenotype Ontology (HPO) terms for participants recruited

In the trio analysis, 143 probands (85 males, 58 females), with severe or syndromic disease, together with their parents, were analysed. They were of different reported ethnicities across England (Fig. 1A).

The age distribution of participants is shown in Fig. 1B. Participants recruited for HCM were more frequent in the 20 to 29 age group, and DCM between ages 20 to 39. LVNC was more common in younger people at enrolment. Of note, participants were enrolled in the 100KGP in their 40 s and 50 s as they still lacked a molecular diagnosis, despite all participants having an age of onset before 40 years of age.

Figure 1C shows the top 20 HPO terms in all participants recruited, including intellectual disability, joint hypermobility, and skeletal myopathy, suggesting syndromic causes.

**Fig. 2:**
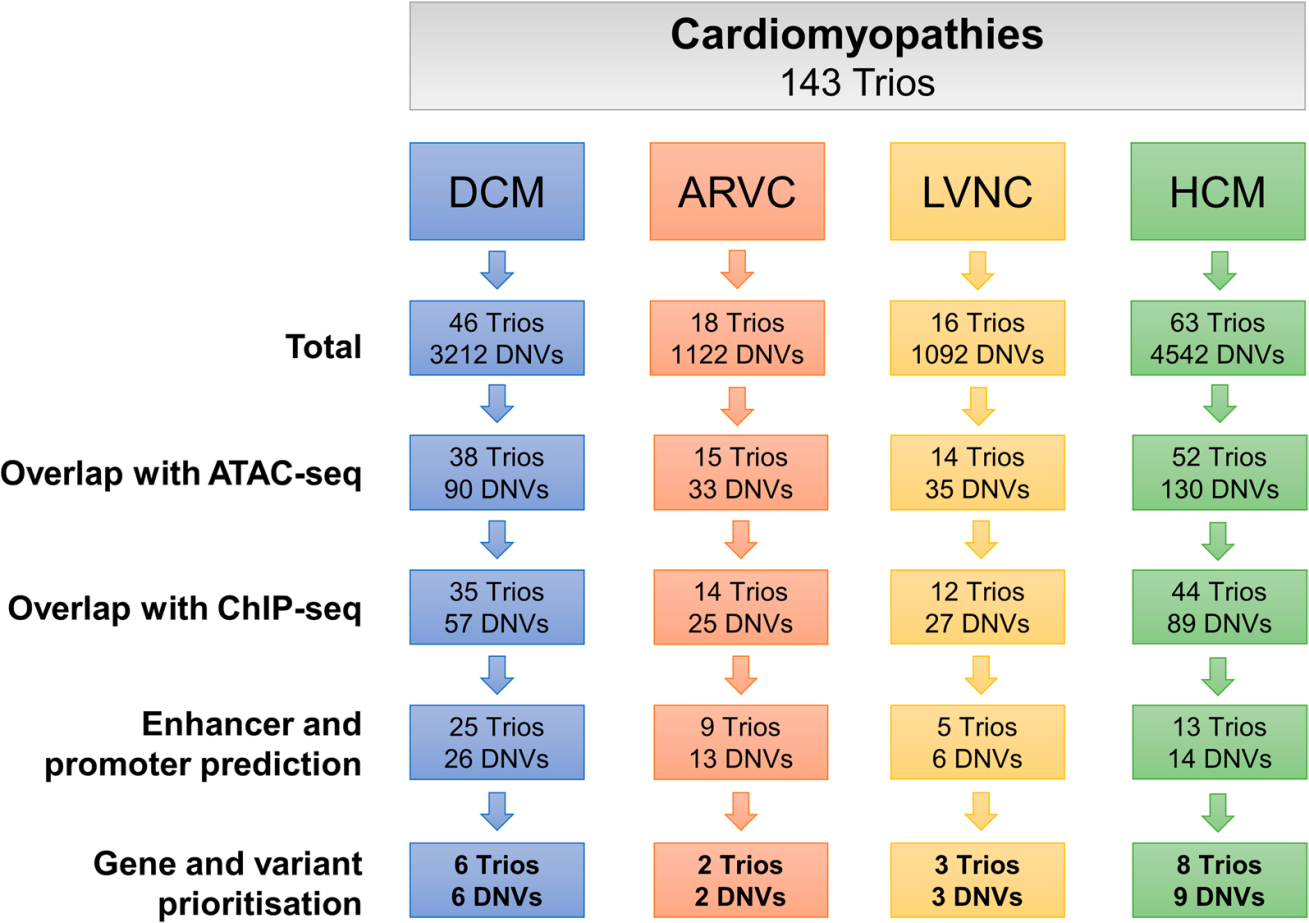
Noncoding de novo variant filtering criteria. DCM = dilated cardiomyopathy; ARVC = arrhythmogenic right ventricular cardiomyopathy; LVNC = left ventricular non-compaction cardiomyopathy; and HCM = hypertrophic cardiomyopathy

After parent–offspring trio analysis and application of our stringent filtering criteria, each proband had an average of 69.7 DNVs (Fig. 2). Genetic findings and genotype–phenotype correlations are described in Tables 1, 2 and 3. We used Exomiser to help narrow down candidate variants.

**Table 1:** De novo coding variants in known disease-risk genes

Family ID	Disease	HPO terms	Genomic position (GRCh38)	Gene	Exomiser score	Consequence	cDNA	Variation	rsID	ClinVar
Fam451	LVNC	Triangular face; astigmatism; downslanted palpebral fissures; intellectual disability; small for gestational age; bicuspid aortic valve; inability to walk; proportionate short stature; abnormal vena cava morphology; proportionate shortening of all digits; feeding difficulties in infant; short proximal phalanx of thumb; low hanging columella; broad hallux; keloids; and delayed fine motor development	chr16:89,280,358 GC > G	*ANKRD11*	0.8794	Frameshift	c.6183del	p.Leu2062TrpfsTer25	–	–
Fam010	HCM	Depressivity; lethargy; palpitations; and pedal oedema	chr2:219,425,734 C > T	*DES*	0.9500	Missense	c.1360C > T	p.Arg454Trp	rs267607490	P/LP
Fam208	DCM	Flexion contracture; congestive heart failure; Achilles tendon contracture; restrictive deficit on pulmonary function testing; skeletal muscle atrophy; progressive muscle weakness; limb muscle weakness; scapular winging; myofibrillar myopathy; skeletal myopathy; knee flexion contracture; limb–girdle muscular dystrophy; and distal lower limb amyotrophy	chr1:156,134,479 T > C	*LMNA*	0.9878	Missense	c.590 T > C	p.Leu197Pro	–	–
Fam520	HCM	Hypothyroidism; congestive heart failure; palpitations; and dyspnoea	chr22:20,987,543 C > A	*LZTR1*	0.8979	Missense	c.360C > A	p.His120Gln	rs1249605552	US
Fam491	HCM	Congestive heart failure; sudden cardiac death; dyspnoea; and arrhythmia	chr14:23,425,285 C > G	*MYH7*	0.9936	Missense	c.2420G > C	p.Arg807Pro	rs141414377	LP
Fam798	HCM	Restrictive cardiomyopathy	chr14:23,429,004 C > T	*MYH7*	0.9209	Missense	c.1358G > A	p.Arg453His	rs397516101	P
Fam268	HCM	Angina pectoris; palpitations	chr14:23,425,970 C > T	*MYH7*	0.9761	Missense	c.2156G > A	p.Arg719Gln	rs121913641	P
Fam659	LVNC	Atrial septal defect; atrioventricular block; asthma; and abnormal ventricular septum morphology	chr5:173,232,976 G > T	*NKX2-5*	0.9684	Missense	c.568C > A	p.Arg190Ser	rs104893906	–
Fam828	HCM	Intellectual disability; failure to thrive; right ventricular cardiomyopathy; and left ventricular non-compaction cardiomyopathy	chr12:112,473,023 A > G	*PTPN11*	0.9609	Missense	c.836A > G	p.Tyr279Cys	rs121918456	P/LP
Fam478	HCM	Palpitations	chr3:12,604,188 G > A	*RAF1*	0.9782	Missense	c.782C > T	p.Pro261Leu	rs397516828	P
Fam794	ARVC	Cardiac arrest; palpitations	chr1:237,756,289 A > G	*RYR2*	0.9958	Missense, splice region	c.11147A > G	p.Glu3716Gly	–	–
Fam231	HCM	Syncope; restrictive cardiomyopathy	chr1:201,365,291 C > T	*TNNT2*	0.8760	Missense	c.311G > A	p.Arg104His	rs397516457	P
Fam112	DCM	–	chr2:178,741,570 C > T	*TTN*	0.9981	Missense	c.11663G > A	p.Gly3888Glu	–	–

The overall Exomiser score (from 0 to 1) is obtained from the variant score and phenotypic score within a logistic regression classifier framework and used for variant prioritisation. Exomiser correctly ranked the causal variant as first in all cases in this list. LP = likely pathogenic; P = pathogenic; and US = uncertain significance

**Table 2:** De novo coding variants in candidate cardiomyopathy genes

Family ID	Disease	HPO terms	Genomic position (GRCh38)	Gene	Exomiser rank	Exomiser score	Consequence	cDNA	Variation	rsID
Fam003	HCM	Ptosis; congestive heart failure; delayed gross motor development; and feeding difficulties	chr19:1,061,809 G > T	*ABCA7*	–	–	Missense	c.5491G > T	p.Ala1831Ser	–
Fam027	DCM	Failure to thrive	chr4:13,376,561 T > C	*RAB28*	–	–	Missense	c.557A > G	p.Glu186Gly	–
Fam043	ARVC	Dilated cardiomyopathy; cardiac arrest; palpitations; abnormal atrioventricular conduction; and arrhythmia	chr19:899,621 G > C	*R3HDM4*	–	–	Stop gained	c.627C > G	p.Tyr209Ter	–
Fam061	HCM	Left ventricular dysfunction	chr1:39,297,744 T > TG	*MACF1*	–	–	Frameshift, splice region	c.2481 + 1dup	–	–
Fam062	LVNC	Failure to thrive; small for gestational age; ventricular septal defect; restrictive cardiomyopathy; hypertrophic cardiomyopathy; dyspnoea; and right ventricular cardiomyopathy	chr12:49,128,718 T > C	*TUBA1B*	1	0.7430	Missense	c.596A > G	p.Asp199Gly	–
Fam137b	DCM	Congestive heart failure; dyspnoea; arrhythmia; and bundle branch block	chr14:77,887,137 C > G	*ADCK1*	3	0.7504	Missense	c.470C > G	p.Ala157Gly	–
Fam137a	DCM	Hypertension; palpitations	chr4:127,764,330 C > T	*SLC25A31*	–	–	Stop gained	c.448C > T	p.Arg150Ter	rs1464153404
Fam190	HCM	–	chrX:118,608,357 G > C	*DOCK11*	2	0.7716	Splice donor	c.2877 + 1G > C	–	–
Fam197	DCM	Syncope; arrhythmia	chr19:48,627,746 C > T	*SPHK2*	1	0.7537	Missense	c.566C > T	p.Pro189Leu	–
Fam225	DCM	Arrhythmia; dyspnoea; nonorganic psychosis; self-harm; eating disorder; and deceased	chr17:41,909,667 G > A	*ACLY*	–	–	Stop gained	c.379C > T	p.Arg127Ter	rs1555633336
			chr15:55,360,123 T > TTTA	*CCPG1*			Inframe insertion	c.1647_1649dup	p.Asn549dup	–
Fam231	HCM	Syncope; restrictive cardiomyopathy; and dyspnoea	chr14:59,263,526 T > C	*DAAM1*	2	0.7490	Missense	c.49 T > C	p.Cys17Arg	–
Fam252	DCM	Failure to thrive; congestive heart failure; and dyspnoea	chr1:110,674,209 C > A	*KCNA3*	1	0.7591	Stop gained	c.601G > T	p.Glu201Ter	–
Fam303	DCM	Eczema; umbilical hernia; and ventricular septal defect	chr1:202,729,832 C > A	*KDM5B*	3	0.7528	Stop gained	c.4372G > T	p.Glu1458Ter	–
Fam313	DCM	Café au lait spot; pulmonic stenosis; dyspnoea; tricuspid regurgitation; right ventricular cardiomyopathy; and dysplastic tricuspid valve	chr5:90,642,886 C > T	*ADGRV1*	3	0.7832	Stop gained	c.2398C > T	p.Arg800Ter	rs373780305
Fam373	HCM	Palpitations; arrhythmia	chrX:136,682,757 C > G	*ARHGEF6*	–	–	Splice donor	c.1479 + 1G > C	–	–
Fam484	LVNC	Intellectual disability; joint hypermobility; Wolff–Parkinson–White; and palpitations	chr15:80,173,016 C > T	*FAH*	–	–	Stop gained, splice region	c.709C > T	p.Arg237Ter	rs769550316
Fam499	HCM	Multifactorial inheritance; familial predisposition; cardiac arrest; and deceased	chr2:240,462,562 G > A	*GPC1*	–	–	Missense	c.697G > A	p.Val233Met	rs748327513
Fam543	HCM	Angina pectoris; restrictive cardiomyopathy	chr22:18,083,639 C > T	*PEX26*	–	–	Stop gained	c.574C > T	p.Arg192Ter	rs61752136
Fam567	HCM	Thin upper lip vermillion; pointed chin; broad forehead; and long palpebral fissure	chr11:47,245,544 G > C	*ACP2*	–	–	Missense	c.479C > G	p.Pro160Arg	–
			chr21:21,418,570 G > T	*NCAM2*	–	–	Splice donor	c.1480 + 1G > T	–	–
Fam571	DCM	Failure to thrive; congestive heart failure; and dyspnoea	chr10:133,186,264 C > T	*KNDC1*	2	0.7507	Stop gained	c.916C > T	p.Gln306Ter	–
Fam659b	LVNC	Atrial septal defect; atrioventricular block; asthma; and abnormal ventricular septum morphology	chr5:141,398,519 C > G	*PCDHGB5*	–	–	Missense	c.392C > G	p.Pro131Arg	rs780671252
Fam728	LVNC	Aortic valve stenosis; aortic regurgitation; and pulmonary insufficiency	chr2:237,344,691 C > T	*COL6A3*	–	–	Missense	c.7327G > A	p.Ala2443Thr	rs1345727801
Fam733	HCM	–	chr15:22,933,819 G > T	*CYFIP1*	1	0.7706	Stop gained	c.975C > A	p.Tyr325Ter	–
Fam806	LVNC	Primum atrial septal defect	chr19:10,143,859 C > T	*DNMT1*	–	–	Missense	c.3023G > A	p.Arg1008Gln	rs746527645
			chr16:17,198,230 G > A	*XYLT1*	–	–	Missense	c.1271C > T	p.Ala424Val	rs148488379
Fam862	DCM	–	chr8:63,026,259 G > T	*GGH*	–	–	Missense	c.398C > A	p.Thr133Lys	–
Fam906	HCM	Hypertension	chr6:98,899,282 G > A	*FBXL4*	–	–	Stop gained	c.1303C > T	p.Arg435Ter	rs201889294

Variants were retained if they had a REVEL score of > 0.5, GERP +  + score > 2, CADD score of > 20 and MAF < 0.001, suggesting high pathogenicity. The overall Exomiser score (from 0 to 1) is shown for variants that were ranked in the top three

**Table 3:** Inherited coding variants in trios in known disease-risk genes

Family ID	Disease	HPO terms	Genomic position (GRCh38)	Gene	Exomiser rank	Exomiser score	Consequence	cDNA	Variation	rsID	Segregation	ClinVar
Fam919	DCM	Microcephaly; congestive heart failure; endocardial fibroelastosis; hydrops fetalis; polymicrogyria; cortical dysplasia; and pedal oedema	chr10:78,009,659 G > A	*POLR3A*	1	0.6862	Missense variant	c.1787C > T	p.Thr596Met	rs756953635	Recessive	–
			chr2:178,549,450 G > A	*TTN*	2	0.3656	Missense variant	c.92176C > T	p.Pro30726Ser	rs72648247	Compound heterozygous	US
Fam599	HCM	Abnormality of the tongue; hypertelorism; wide nasal bridge; preaxial hand polydactyly; intellectual disability; congestive heart failure; neonatal onset; prominent epicanthal folds; abnormal social behaviour; and mild microcephaly	chr11:47,342,698 G > A	*MYBPC3*	–	–	Missense variant	c.1504C > T	p.Arg502Trp	rs375882485	Dominant	P
Fam099	HCM	–	chrM:6120 A > G	*MT-CO1*	–	–	Missense variant	c.217A > G	p.Ile73Val	rs878853023	Mitochondrial	US
Fam478	HCM	Abnormality of weight; spare scalp hair; frontal bossing; congestive heart failure; dry skin; and deeply set eye	chrM:14,279 G > A	*MT-ND6*	–	–	Missense variant	c.395C > T	p.Ser132Leu	rs869025187	Mitochondrial	P
Fam180	HCM	–	chr14:23,429,094 G > A	*MYH7*	1	0.9975	Missense variant	c.1268C > T	p.Ala423Val	rs1177694963	Dominant	–
Fam484	LVNC	Intellectual disability; joint hypermobility; Wolff–Parkinson–white syndrome; and palpitations	chr7:93,103,844 A > T	*SAMD9*	–	–	Missense variant	c.2254 T > A	p.Trp752Arg	rs148339415	Dominant	–
Fam992	ARVC	–	chr18:31,524,723 AAATC > A	*DSG2*	–	–	Frameshift variant	c.852_855del	p.Asn284LysfsTer4	rs1165139589	Dominant	–
			chr18:31,531,006 TGAA > T	*DSG2*	–	–	Inframe deletion	c.1038_1040del	p.Lys346del	rs727502987	Dominant	–
Fam539	HCM	Skeletal myopathy; increased nuchal translucency	chr21:45,999,190 A > C	*COL6A1*	–	–	Missense variant	c.1712A > C	p.Lys571Thr	rs751040647	Dominant	US
			chr22:20,993,712 G > A	*LZTR1*	3	0.1816	Stop gained	c.1311G > A	p.Trp437Ter	rs770933647	Dominant	P
			chr2:178,725,987 T > A	*TTN*	–	–	Missense variant	c.20335A > T	p.Ser6779Cys	rs149470241	Compound heterozygous	US
Fam957	DCM	Partial anomalous pulmonary venous return; dyspnoea; myocardial fibrosis; and oligospermia	chr15:34,793,327 G > C	*ACTC1*	1	0.8597	Missense variant	c.372C > G	p.Ile124Met	rs397517061	Dominant	US
Fam411	HCM	Palpitations	chr11:47,352,622 C > T	*MYBPC3*	2	0.9375	Splice donor variant	c.25 + 1G > A	–	rs113709679	Dominant	P

The overall Exomiser score (from 0 to 1) is shown for variants that were ranked in the top three. P = pathogenic; US = uncertain significance

In addition, 843 exome-negative cardiomyopathy probands and 19,467 controls were incorporated into our case-control study from the 100KGP. The cases are singleton offspring whose parental WGS data were unavailable. Using the PanelApp software, which contains a crowd-sourced curation of genes with diagnostic-grade evidence, only cases lacking a molecular diagnosis were recruited in the case–control analysis. These cases included 61.8% HCM, 26.0% DCM, 8.5% ARVC, and 3.6% LVNC subtypes. Most participants were of European ancestry (70%), and 64% were male.

De novo variants in diagnostic-grade genes.

Using the American College of Medical Genetics (ACMG) guidelines, we identified deleterious DNVs in 11/143 trios (7.7%). These class 4 and 5 variants are defined as likely pathogenic or pathogenic, and are reported as consistent with or confirming a diagnosis, respectively (Table 1). Exomiser ranked the correct diagnosed variants as the top candidate in all these cases, and no parents were affected. Several novel DNVs were identified in syndromic and non-syndromic cases.

In Fam659, the proband had a novel missense DNV c.568C > A (p.Arg190Ser) in the *NKX2-5* gene. *NKX2-5* (NK2 homeobox 5) encodes for a transcription factor that is important for the development of the myocardium [27]. Mutations in this gene are known to cause congenital heart disease, particularly atrial septal defect (with or without atrioventricular conduction defects), and ventricular septal defect. Consistent with this, the patient was diagnosed with LVNC, including atrial septal defect, atrioventricular block, and abnormal ventricular septum morphology.

In the proband of Fam208, a novel missense DNV c.590 T > C (p.Leu197Pro) was identified in the *LMNA* gene (lamin A/C). *LMNA* encodes the A-type lamin proteins, lamin A and C, which are the major components of the nuclear membrane in mammals. Mutations in *LMNA* have been reported to cause a variety of clinical phenotypes, collectively known as laminopathies. These include cardiac disorders, premature ageing syndromes, and neuropathies [28]. In addition to DCM, the proband had a range of musculoskeletal-related abnormalities (Table 1).

In the proband of Fam520, a missense DNV c.360C > A (p.His120Gln) was found in the *LZTR1* gene. Mutations in *LZTR1* (leucine-zipper-like transcriptional regulator 1) are associated with Noonan syndrome phenotypes and schwannomatosis [29]. As well as obstructive HCM, the proband had combined disorders of mitral, aortic and tricuspid valves, congestive heart failure, thyrotoxicosis with diffuse goitre, postprocedural hypothyroidism, and rheumatoid arthritis. According to the ACMG guidelines, the variant is classified as a variant of uncertain significance. It is predicated to be deleterious according to in silico algorithms and was not identified in public databases, including gnomAD and 1000G. We require further clinical information to determine a phenotype consistent with Noonan spectrum disorders. Dominant and recessive Noonan syndrome-causing mutations have been described near this variant, all of which within the highly conserved kelch domains [30, 31].

A novel frameshift DNV of high-impact c.6183del (p.Leu2062TrpfsTer25) in the *ANKRD11* gene was observed in the proband of Fam451. Mutations in *ANKRD11* (ankyrin repeat domain 11) are reported to be associated with KBG syndrome and intellectual disability [32]. The patient presented with LVNC and a variety of other phenotypes, including abnormal vena cava morphology, bicuspid aortic valve, abnormality of the face, delayed fine motor development, intellectual disability, and proportionate short stature (Table 1).

In the proband of Fam478, a known pathogenic missense DNV c.782C > T (p.Pro261Leu) was found in the *RAF1* gene. Diseases associated with *RAF1* (Raf1 proto-oncogene, serine/threonine kinase) include Noonan and Leopard syndromes. These developmental disorders have overlapping features, including cardiac abnormalities, short stature, and facial dysmorphia [33]. The proband was diagnosed with HCM and had other phenotypes, including congenital malformation of cardiac septum, palpitations, anxiety disorder, and depression. This DNV has previously been reported in an individual affected with Noonan syndrome, including other individuals with clinical features of this disease [33–35]. Moreover, functional studies have shown that p.Pro261Leu leads to increased activity of the RAF1 protein [36].

In the proband of Fam828, we found a missense DNV c.836A > G (p.Tyr279Cys) in the *PTPN11* gene (protein tyrosine phosphatase non-receptor type 11). Mutations in *PTPN11* are well characterised in children with Noonan syndrome and juvenile myelomonocytic leukaemia [37]. In addition to HCM, the proband had other phenotypes, including intellectual disability, failure to thrive, right ventricular cardiomyopathy, and LVNC.

Other known pathogenic DNVs were found consistent with a diagnosis of non-syndromic cardiomyopathy. Three participants with HCM had known missense variants in *MYH7*. Two variants (c.2156G > A (p.Arg719Gln) and c.1358G > A (p.Arg453His)) are reported as pathogenic on ClinVar, whereas variant c.2420G > C (p.Arg807Pro) is reported as likely pathogenic. The proband harbouring the latter *MYH7* variant also had congenital malformations of the heart, congestive heart failure, arrhythmia, and died as an infant with sudden cardiac arrest. Pathogenic variants were found in other known genes, including *DES*, *RYR2*, *TTN* and *TNNT2* (Table 1).

### De novo variants in other disease-risk genes

We also looked at DNVs in genes not included in PanelApp. In 27/143 probands (18.9%), 30 rare de novo coding variants were identified, which were considered deleterious based on in silico prediction tools (see methods). Using Exomiser, a phenotype-based prioritisation pipeline, 11 DNVs were ranked in the top three as the most likely cause. These include *TUBA1B, KIRREL1, DAAM1, DOCK11*, and *KDM5B* (Table 2). It is possible that some of the putative genes identified herein could be novel gene candidates or genetic modifiers.

Interestingly, the proband of Fam231 harbouring a missense DNV c.49 T > C (p.Cys17Arg) in the *DAAM1* gene also had a known pathogenic variant in *TNNT2* (Table 1). Studies show that *DAAM1* is required for cardiomyocyte maturation [38], and deletion of the gene is associated with congenital heart anomalies [39]. Multiple gene mutations occurring in cardiomyopathy families may result in a more severe clinical phenotype because of a compounding effect. Other examples of oligogenic inheritance are described below.

### Inherited variants in diagnostic-grade genes

In 10/143 trios (7.0%), 14 rare inherited variants of potential clinical significance were identified in probands and their affected family members based on gene panel testing. These include variants with recessive, dominant, and compound heterozygous segregation patterns (Table 3).

Two participants with HCM had missense variants in *MYBPC3*. In Fam599, the proband harbouring the c.1504C > T (p.Arg502Trp) variant presented with a range of diagnoses, including abnormal thumb, eye and oral morphology, intellectual disability, and mild microcephaly. The mother, also harbouring the variant, was diagnosed with atrial septal defect, short thumb, polycystic ovaries, and Raynaud syndrome. It is not clear why the proband presented with a more severe phenotype.

In Fam411, the proband inherited the *MYBPC3* splice donor variant c.25 + 1G > A from the affected mother. In addition to HCM, the mother presented with hypothyroidism, prolonged QT syndrome, severe depression, anxiety, and schizoid personality disorder.

In Fam484, the proband inherited an autosomal dominant mutation c.2254 T > A in *SAMD9* from the affected father. The proband was diagnosed with LVNC, ventricular septal defect, intellectual disability, joint hypermobility, and Wolff–Parkinson–White syndrome. Although the affected father and sibling carrying this variant presented with LVNC, neither had the comorbidities present in the proband. The father, however, presented with gastrointestinal haemorrhage, and diverticular disease of large intestine (without perforation or abscess). Mutations in *SAMD9* have been described in patients with MIRAGE syndrome, a severe multisystem disorder [40]. This includes prominent gastrointestinal symptoms and intellectual disability.

In the proband and affected mother of Fam957, a missense variant c.372C > G (p.Ile124Met) was identified in the *ACTC1* gene. Mutations in *ACTC1* (actin alpha cardiac muscle 1) are associated with atrial septal defect, DCM, and HCM [41, 42]. In addition to DCM, the proband had partial anomalous pulmonary venous return, dyspnoea, myocardial fibrosis, and oligospermia. The mother was diagnosed with DCM, secundum atrial septal defect, and bipolar affective disorder.

Multilocus inheritance may explain the relatively low diagnostic yield for cardiomyopathy cases, or apparent phenotypic expansion. We found evidence for compound heterozygosity, digenic, and oligogenic inheritance in several families (described below). This highlights the importance of screening for additional genes even after a single mutation has been identified.

We observed evidence for oligogenic inheritance in Fam919. The proband, born in 2018 and reported to be deceased, had a recessive mutation in *POLR3A* (c.1787C > T (p.Thr596Met)) and a compound heterozygous *TTN* mutation (c.92176C > T (p.Pro30726Ser)). Mutations in *POLR3A* are associated with a wide array of pathological phenotypes, some of which were present in the proband. In addition to DCM and congestive heart failure, the proband had multiple congenital anomalies, including microcephaly, endocardial fibroelastosis, hydrops fetalis, polymicrogyria, cortical dysplasia, and pedal oedema.

The proband of Fam539 inherited five compound heterozygous *TTN* variants, one of which from the mother passed our filtering criteria for deleteriousness (c.20335A > T, p.Ser6779Cys) (see methods). The proband also inherited autosomal dominant variants of incomplete penetrance in *COL6A1* and *LZTR1*. The *LZTR1* stop gained variant c.1311G > A (p.Trp437Ter) was inherited from the mother, and the *COL6A1* missense variant c.1712A > C (p.Lys571Thr) was inherited from the father. The proband was diagnosed with HCM, skeletal myopathy, and increased nuchal translucency. One out of two siblings are also affected with HCM; however, detailed medical notes or WGS data are not available. Although considered unaffected for cardiomyopathy, both parents were diagnosed with primary (essential) hypertension. In addition, the father has a family history of ischaemic heart disease, arrhythmia, syncope and collapse, and other ill-defined heart diseases.

In the proband of Fam992, compound heterozygous mutations c.852_855del (p.Asn284LysfsTer4) and c.1038_1040del (p.Lys346del) in the *DSG2* gene were identified. The mother of the proband had the frameshift variant c.852_855del, whereas the father had the inframe deletion c.1038_1040del. In addition to ARVC, the proband was diagnosed with disorders of magnesium metabolism, hypokalaemia, and congenital malformations of cardiac chambers and connections. Of note, a DNV in the enhancer of gene *TUSC3* was also identified. *TUSC3* constitutes a major component in cellular magnesium transport and homeostasis, and its function in regulation of embryonic development in vertebrates has been suggested [43, 44]. This may explain the disorders of magnesium metabolism and hypokalaemia as a secondary cause in the proband [45].

In Fam180, the proband and mother with HCM harbour a variant of unknown significance (class 3) in the *MYH7* gene. In addition, two families with HCM had mutations in mitochondrial genes *MT-CO1* and *MT-ND6* (Table 3); both previously implicated in heart disease, although heteroplasmy proportions are yet to be determined in multiple tissue samples.

These examples demonstrate that non-Mendelian inheritance may be an important factor in the cardiomyopathy cause-discovery pipeline. Other possibilities exist to help close the diagnostic gap, including noncoding mutations that affect regulatory elements.

### Singe-cell chromatin state profiling

We hypothesised that DNVs in human heart regulatory regions are more likely to perturb expression levels of genes that are essential for cardiac function.

Annotation of ventricular cardiomyocyte peak set in genomic features shows enrichment in intronic and distal intergenic regions and in the flanking regions of TSSs, suggesting an enrichment of gene regulatory elements, such as enhancers. We intersected single-cell ATAC-seq peaks with publicly available H3K27ac ChIP-seq data (a marker for active enhancers) of eight healthy adult donors [46] and found significant overlap with our peaks (Permutation test, one-sided, *p* < 0.001).

### Identification of de novo variants in noncoding regulatory regions

To determine selective vulnerabilities across diverse cell types of the human heart, we intersected cell-type-specific ATAC-seq peaks with DNVs identified from parent–offspring trio analysis (see methods). Cardiomyocyte-specific peak sets were not significantly enriched for DNVs in offspring compared to a merged background peak set.

A total of 288 DNVs from 143 trios intersected with a peak signal from ventricular cardiomyocytes. After filtering for parental affected status, H3K27ac overlap, and mapping regulatory regions to genes, 15 DNVs were within promoter regions, and 12 within predicted enhancers linked to their target genes. We used the tool FATHMM-MKL to predict the functional effects of noncoding variants. Additional file 1: Table S1 shows prioritised variants within ventricular cardiomyocyte open chromatin regions.

### Predicting the target genes of enhancers

Using the ABC model [18], we predicted likely enhancers by integrating H3K27ac ChIP-seq, HiChIP, and gene expression data with chromatin accessibility. We identified sets of high-confidence putative enhancers for ventricular cardiomyocytes and their likely target genes.

As a complementary approach, histone ChIP-seq experiments on Hi-C samples were analysed to provide epigenetic features using 3DIV. Annotation of enhancer/super-enhancers and histone ChIP-seq signals were provided for the following: H3K27ac, H3K27me3, H3K36me3, H3K4me1, H3K4me3. Genes with distance-normalised interaction frequency > 2 were retained. In addition, we used promoter capture Hi-C data to detect interactions with gene promoters. These data are summarised in Additional file 1: Table S1.

### Network analysis

We applied another strategy to further prioritise the effect of DNVs on human cardiac regulome. We analysed the 62 genes associated with enhancers and promoters containing prioritised DNVs using VarElect to correlate their functions with different aspects of the clinical phenotype. Results suggest that 20 targets were directly related to cardiomyopathy, whereas 41 were indirectly related (Additional file 2: Table S2). One gene (*MIR3143*) was unrelated and therefore excluded from the analysis. Among the unified results, the top five genes with the highest score of correlation were *JPH2, UTRN, H1-2, RHOD, and SAP30B*. This score is an indication of the strength of the connection between the gene and the queried phenotypes. The score helps to rank and prioritise the list of queried genes by relevance to the disease. Interestingly, many of the top scoring genes were associated with the same DNV.

### Noncoding de novo variants are associated with cardiomyopathy-risk genes

**Fig. 3:**
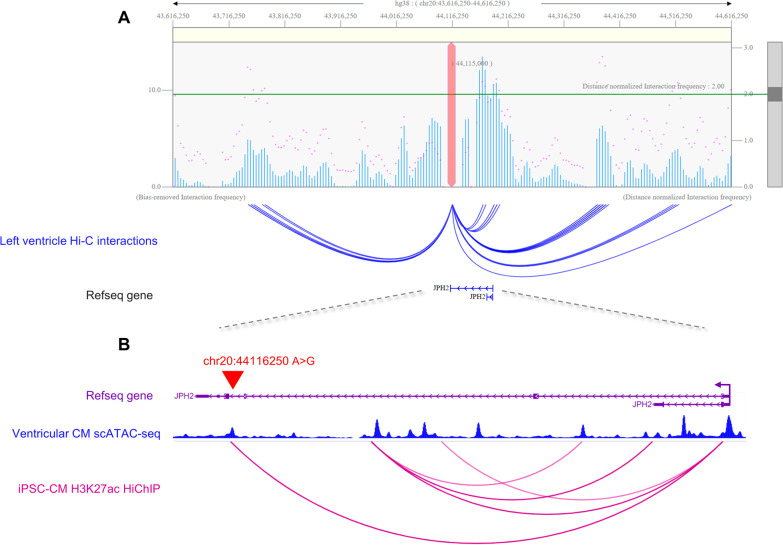
Chromatin interaction map of chr20:44,116,250 locus. **A** One-to-all interaction plots are shown for the lead variant chr20:44,116,250 A > G shown in blue as bait. Y-axes on the left and the right measure bias-removed interaction frequency (blue bar graph) and distance-normalised interaction frequency (magenta dots), respectively. The arc representation of significant interactions for distance-normalised interaction frequencies ≥ 2 is displayed relative to the Refseq-annotated genes in the locus. The DNV physically interacts with the *JPH2* enhancer by long-range chromatin interaction. **B** Close-up of region containing *JPH2*. The ABC model independently predicted the DNV (red triangle) is within the *JPH2* enhancer. CM = cardiomyocyte

In the proband of Fam499, we identified a DNV within an enhancer of *JPH2* (Fig. 3). This gene exhibited the highest phenotype association (VarElect score 36.89) (Additional file 2: Table S2). The proband, female and of British ethnicity, was diagnosed with HCM and reported to have died due to sudden cardiac arrest in the year 2020, at the age of 19. Both parents and natural sibling recruited in the study were unaffected.

The junctophilin-2 gene (*JPH2*) is the major structural protein in cardiomyocytes for coupling of transverse tubule-associated L-type Ca^2+^ channels and type-2 ryanodine receptors on the sarcoplasmic reticulum within junctional membrane complexes (JMC) [47]. Signalling between these two Ca^2+^ channels is required for normal cardiac contractility. Disruption of the JMC is a common finding in failing hearts. Downregulation of *JPH2* gene has been associated with heart failure, and mutations in this gene are associated with HCM [47, 48].

*JPH2* was the only high-evidence gene found in our noncoding DNV analysis. We recognise that caution be exercised in the interpretation of variants in cardiomyopathy genes lacking robust evidence; however, the following preliminary results may help to explain the complex genetic architecture of cardiomyopathy.

The proband of Fam126 was diagnosed with DCM and harbours a DNV within an enhancer region that regulates the genes *UTRN*, *STX11*, and *SF3B5*. Diseases associated with *UTRN* (utrophin) include muscular dystrophy, endothelial dysfunction, and DCM [49, 50].

The proband of Fam313 was diagnosed with DCM, including dysplastic tricuspid valve, right ventricular cardiomyopathy, tricuspid regurgitation, dyspnea, pulmonic stenosis, café au lait spot, and congenital heart disease. Although no DNVs were identified in enhancer regions, a deleterious coding DNV was identified in *ADGRV1* (Table 2). In addition, we used pcHi-C to detect distal promoter-interacting regions and found that a DNV in this proband is associated with a large cluster of histone genes on human chromosome 6.

Studies have shown that histone acetylation/deacetylation regulates cardiac morphogenesis, growth, and contractility [51]. Gene expression profiles of DCM patients have also shown that several histone family members are downregulated [52]. We hypothesise that the downregulation of these genes, which are responsible for higher order chromatin structure, may contribute to the clinical presentation in this proband via nucleosome formation blockade [53].

The proband of Fam334 was initially diagnosed with DCM, and later suspected to have aortopathy and early hypertension. Other diagnoses include lethargy, torsion of testis, headaches, and palpitations. The proband died in the accident and emergency department in the year 2018, at the age of 21. Neither parent or three natural siblings were affected. The DNV identified in this proband is (1) within the enhancer region of genes *UNC13D, WBP2, SAP30BP* and *TRIM65*; (2) overlapping the promoter regions of genes *H3-3B*, *MIR4738* and *UNK*; and (3) interacting with distil gene promoters of *TRIM56* and *TMEM94*. Diseases associated with *TMEM94* include cardiac defects [54].

In Fam791, the proband was diagnosed with ARVC and DCM, in addition to arrhythmia, varicose veins of lower extremities with ulcer and inflammation, atherosclerotic heart disease, renal failure, gastrointestinal haemorrhage, and anal polyp. A DNV was identified within the enhancer region of genes *GRK2* and *RHOD*, and overlapping the promoter region of *RAD9A*.

G protein-coupled receptor kinase-2 (GRK2) regulates many cellular and physiological processes, including cardiac contractility, cell proliferation, cell cycle regulation, angiogenesis and vasodilatation. Inhibiting GRK2 can enhance cardiac contractility and protect from adverse heart remodelling in disorders related to cardiac dysfunction [55], suggesting its inhibition as a therapeutic strategy for heart failure. We hypothesise that this DNV may elevate levels and activity of this kinase, thus promoting cardiovascular disease. Moreover, phenome-wide associated loci in the proximity of *RHOD* is a likely causal gene for cardiomegaly and hematemesis [56], the latter of which may explain the gastrointestinal bleeding observed in this patient.

### Case-control analysis reveals high-risk noncoding variants in disease-risk genes

The contribution of disease-causing rare variants in noncoding regulatory regions remains elusive. The identification of candidate noncoding DNVs in our trio analysis led us to investigate high-risk regulatory variants associated with cardiomyopathy genes in a large cohort lacking a pathogenic mutation in gene panel testing.

Overall, combining data from all genes, there was a significant difference in the proportion of cardiomyopathy cases and controls carrying one or more rare variant in regulatory elements of strong or definitive disease-risk genes (p = 0.035, OR = 1.43, 95% Cl = 1.095–1.767).

Of the 843 probands lacking a molecular diagnosis, we performed variant-level analysis and identified 25 noncoding variants that were significantly associated in cases (*p*< 0.05). Of these, 9 predicted to effect TF binding motifs. Eight of the 12 genes investigated had one or more rare variant in regulatory regions, including *DSP, RBM20, LMNA, TNNT1, TNNT2*, *BAG3, DES*, and *PLN*. The highest-ranking regulatory elements for each gene are listed in Additional file 3: Table S3.

Most of the significant variants (n = 23; 92%) were “private” to a single proband, with only two variants occurring in two unrelated probands, albeit with the same cardiomyopathy subtype. The private variants were not identified in control populations. Most variants (76%) occurred in HCM, 12% occurred in DCM, 8% occurred in ARVC, and 4% were observed in LVNC cases. These data are summarised in Table 4.

**Table 4:** Rare noncoding variants identified in regulatory elements of definitive cardiomyopathy genes in the case-control analysis

Proband ID	Disease	Genomic position (GRCh38)	Gene	MAF Controls	*p* value	FATHMM-MKL score	Motifs
P01	HCM	chr1:156,106,052 G > T	*LMNA*	0	0.0415	0.08434	
P02	HCM	chr1:156,106,161 T > C	*LMNA*	0	0.0415	0.05647	
P03	DCM	chr1:156,106,287 T > G	*LMNA*	0	0.0415	0.39668	Bcl6, EHF, ELF1, ELF3, ELF4, ELF5, STAT1, STAT3, Stat4, Stat5a::Stat5b
P04	DCM	chr1:156,106,347 T > G	*LMNA*	0	0.0415	0.31391	
P05	DCM	chr1:156,106,457 C > T	*LMNA*	0	0.0415	0.54018	
P06	HCM	chr1:201,377,783 C > A	*TNNT2*	0	0.0415	0.22864	PLAG1
P07; P08	HCM; HCM	chr1:201,377,789 G > A	*TNNT2*	2.57E-05	0.005	0.19213	PLAG1, RREB1, ZNF263
P09	HCM	chr1:201,377,790 A > G	*TNNT2*	0	0.0415	0.15782	RREB1
P10, P11	HCM; HCM	chr10:110,637,018 C > A	*RBM20*	7.71E-05	0.0158	0.56143	
P12	HCM	chr10:119,651,128 G > C	*BAG3*	0	0.0415	0.65553	
P13	LVNC	chr10:119,651,207 G > C	*BAG3*	0	0.0415	0.66491	HINFP
P14	HCM	chr10:119,651,329 T > C	*BAG3*	0	0.0415	0.54331	EGR3, EGR4, SP2
P15	HCM	chr10:119,651,409 T > C	*BAG3*	0	0.0415	0.98892	
P16	HCM	chr2:219,419,925 C > T	*DES*	0	0.0415	0.89101	
P17	ARVC	chr2:219,419,965 C > G	*DES*	0	0.0415	0.26643	
P18	HCM	chr3:52,452,725 T > G	*TNNC1*	0	0.0415	0.89566	CTCF, Hic1, HIC2, Myod1, SNAI2
P19	HCM	chr6:7,541,668 G > C	*DSP*	0	0.0415	0.36401	
P20	HCM	chr6:7,542,061 C > T	*DSP*	0	0.0415	0.20036	
P21	HCM	chr6:7,542,062 C > G	*DSP*	0	0.0415	0.38808	
P22	HCM	chr6:7,542,072 T > C	*DSP*	0	0.0415	0.90083	
P23	ARVC	chr6:7,542,155 G > C	*DSP*	0	0.0415	0.1913	
P24	HCM	chr6:7,542,266 G > C	*DSP*	0	0.0415	0.19228	
P25	HCM	chr6:118,537,675 A > C	*PLN*	0	0.0415	0.89431	Nr1h3::Rxra
P26	HCM	chr6:118,537,684 T > C	*PLN*	0	0.0415	0.90162	Nr1h3::Rxra
P27	HCM	chr6:118,537,778 A > G	*PLN*	0	0.0415	0.22901	

*P* values are calculated using the Fisher’s exact test. Motifs column indicate functional motifs present according to the funMotifs framework. The FATHMM-MKL score indicates the pathogenic impact of individual SNVs. Predictions are given as p values in the range 0 to 1; values > 0.5 are predicted to be deleterious

## Discussion

The pathogenesis of cardiomyopathy is largely unknown, and the diagnosis is challenging due to its clinical heterogeneity, involving incomplete penetrance and variable expression. The analysis of DNVs in clinically well-defined phenotypes is a powerful approach to delineate the aetiological basis of disease as it focuses on a relatively small number of variants that provide strong evidence of pathogenicity [57].

DNVs are responsible for the relatively high prevalence of complex disorders. The estimated rate for human germline de novo SNVs is (1.0 to 2.4) × 10^−8^ per base per generation [58, 59]. This translates to an average of 32 to 76.8 variants in the human genome, with one or two in exonic regions. We had an average of 69.7 DNVs per trio analysis, giving a mutation rate of 2.2 × 10^−8^ per base per generation. This is consistent with previous studies, thus showing the high quality of our data.

We combined gene panel testing with Exomiser, a phenotype-based algorithmic framework, to prioritise inherited and DNVs. A definite or probable genetic diagnosis was identified in 21 probands according to the ACMG guidelines. Additional DNVs of potential clinical significance were identified in 30 genes, 11 of which were within the top three ranked by Exomiser, including *TUBA1B, KIRREL1, DAAM1, DOCK11*, and *KDM5B*.

In addition, we integrated WGS and single-cell epigenomics to examine the role of regulatory DNVs in cardiomyopathies. Despite the genetic heterogeneity of cardiomyopathy, which stifles efforts to unequivocally demonstrate a causal role for individual noncoding variants, our results provide multiple lines of evidence to indicate the aetiological basis of functional regulatory variants in the human heart regulome. Notably, a DNV was identified within an enhancer of *JPH2,* a gene associated with HCM and the highest scored in our analysis.

Interestingly, we found that more than one rare variant in different cardiomyopathy genes may be relevant for disease causation. Other studies have shown that cardiomyopathy can arise from co-inheritance of rare genetic variants that are benign on their own but harmful in combination [60]. The assumption that all or most patients will receive a single-gene diagnosis is now relegated to the margins. Investigating additional affected families does not necessarily lead to novel gene discovery, thus necessitating the exploration of non-Mendelian contributors to causation or risk.

To further add weight to the hypothesis that noncoding variants are associated with cardiomyopathy, we performed a case-control analysis in a mutation-negative cohort and found an enrichment of high-impact regulatory SNVs in cases compared to controls. A variant-level association test showed that 25 SNVs were significantly associated with disease, of which 23 were not identified in control populations and nine are predicted to alter TF motifs.

There were several limitations in this study. It is possible that probands in the trio analysis inherited variants within noncoding loci associated with disease, or inherited coding variants in genes beyond those listed in the applied panels. Moreover, in the case–control analysis, we only focused on genes that are strongly associated with cardiomyopathy. We also did not analyse structural variants, such as CNVs, inversions, balanced translocations, or complex rearrangements.

**Fig. 4:**
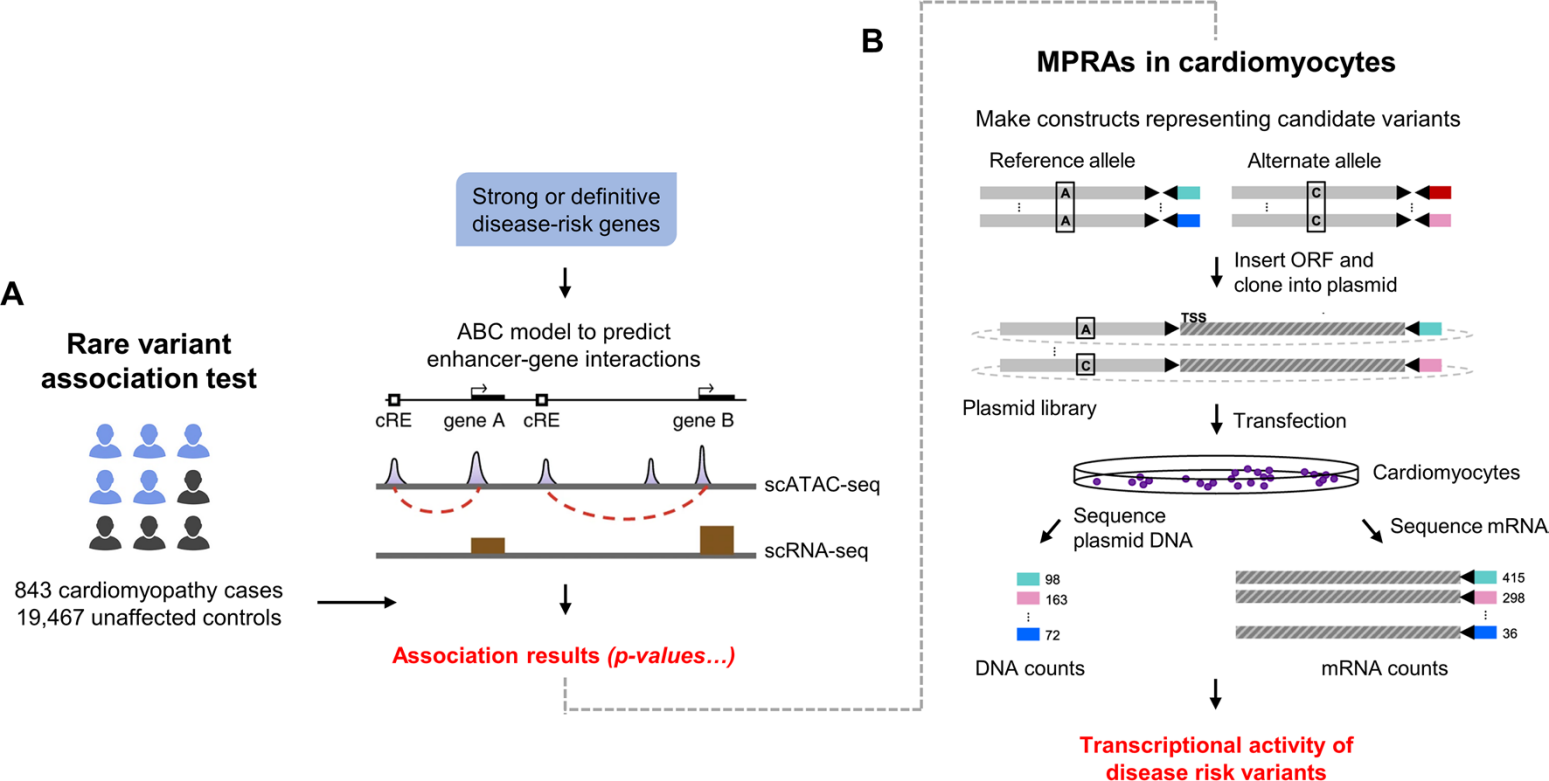
An integrative genomics approach for prioritising noncoding variants. **A** We performed a rare-variant association study in cardiomyocyte-specific regulatory regions of genes associated with cardiomyopathy. These variants should be tested for deleteriousness or transcriptional activity, and, by inference, for causality. **B** MPRAs allow for thousands of short DNA sequences to be assayed simultaneously by first synthesising DNA oligos on an array, integrating them into plasmids and inserting into cells. Both input DNA and RNA libraries are sequenced to assess the tag counts associated with the test sequences. Barcode abundance thus scales quantitatively with the regulatory activity of a given tested sequence (figure adapted from Ajore et al. [63]). This technique can be used in future studies to screen all prioritised cardiomyocyte-specific regulatory variants in cardiomyopathy cases

Indeed, functional validation of the novel variants reported herein is warranted, a lack of which is acknowledged as a further limitation. Novel variants should not be considered causal merely because they are rare and predicted to be deleterious *in silico* [61]. Many of the disease-associated variants identified in this study are noncoding, which are in less-well understood regions of the genome. High-throughput assays with functional readout for putative regulatory elements would enable the identification of functional variants and the biological contexts in which they act. Massively parallel reporter assays (MPRAs) permit the high-throughput functional characterisation of noncoding genetic variation [62]. In Fig. 4, we offer a workflow to identify candidate noncoding variants associated with disease, and to assess the molecular consequences of their disruption experimentally. Adapting MPRAs for use in cardiomyocytes will be critical towards understanding cell-type-specific models of regulatory logic in contexts of greater clinical relevance.

Our work brings together multiple ‘omics datasets to elucidate the role of pathogenic variants in coding and noncoding loci. This study should prompt extensive genetic analyses and variant-specific experimental modelling to elucidate the complex genetic mechanisms underlying cardiomyopathies.

## Supplementary Information

**Additional file 1: Table S1:** Prioritised noncoding DNVs within the human ventricular cardiomyocyte regulome.

**Additional file 2: Table S2:** All gene targets of noncoding DNVs directly or indirectly associated with cardiomyopathy.

**Additional file 3: Table S1:** Activity-by-Contact (ABC) model predictions of known cardiomyopathy genes in ventricular cardiomyocytes.

## Data Availability

All datasets generated for this study are included in the article and supplementary files. Primary data from the 100KGP database are held in a secure Research Environment and available to registered users. Requests to access the primary data should be directed to https://www.genomicsengland.co.uk/about-gecip/for-gecip-members/data-and-data-access. Further information about the data and conditions for access are available from the University of Northampton Research Explorer at: http://doi.org/10.24339/87f60dbe-40e9-4f3e-bcd3-f5d072575431.
