# Correction: De novo and inherited variants in coding and regulatory regions in genetic cardiomyopathies

**DOI:** 10.1186/s40246-025-00825-7

**Published:** 2025-10-24

**Authors:** Nirmal Vadgama, Mohamed Ameen, Laksshman Sundaram, Sadhana Gaddam, Casey Gifford, Jamal Nasir, Ioannis Karakikes, Genomics England Research Consortium

**Affiliations:** 1 Department of Cardiothoracic Surgery and Cardiovascular Institute, Stanford University, 240 Pasteur Drive, Palo Alto, CA 943054 USA; 2 Department of Pediatrics, Division of Cardiology, Stanford School of Medicine, Lucile Packard Children’s Hospital, Palo Alto, CA USA; 3 Department of Genetics, Stanford School of Medicine, Lucile Packard Children’s Hospital, Palo Alto, CA USA; 4 BASE Initiative, Betty Irene Moore Children’s Heart Center, Lucile Packard Children’s Hospital, Palo Alto, CA USA; 5 Department of Cancer Biology, Stanford University, Stanford, CA USA; 6 Department of Computer Science, Stanford University, Stanford, CA USA; 7 Program in Epithelial Biology and Department of Dermatology, Stanford University School of Medicine, Stanford, CA USA; 8 Genomics England, London, UK; 9 Division of Life Sciences, University of Northampton, Waterside Campus, University Drive, Northampton, NN1 5PH UK

**Correction: Human Genomics (2022) 16:55** 10.1186/s40246-022-00420-0

The article has been updated to remove identifying information.
