# Supplementary material for: De novo and inherited variants in coding and regulatory regions in genetic cardiomyopathies
Source: Hum Genomics. 2022 Nov 10;16:55. doi: 10.1186/s40246-022-00420-0 (PMC9647983; doi:10.1186/s40246-022-00420-0)
Supplement: Supplementary file 1 — Additional file 1: Table S1: Prioritised noncoding DNVs within the human ventricular cardiomyocyte regulome. [file 40246_2022_420_MOESM1_ESM.pdf]

Table S1: Prioritised noncoding DNVs within the human ventricular cardiomyocyte regulome. We used the ABC model to predict enhancer–gene connections in cardiomyocytes. Promoters were defined as 2 kb upstream or 1 kb downstream of TSSs. Promoter Capture Hi-C (pcHi-C) interactions were obtained from the 3DIV database. The Fathmm-MKL score indicates the pathogenic impact of individual SNVs. Predictions are given as p-values in the range 0 to 1; values > 0.5 are predicted to be deleterious.

Family ID	Disease	Genomic position (GRCh38)	ABC predictions	-2kb/1kb of TSS	3DIV pcHi-C	Fathmm-MKL score
Fam000	HCM	chr8:140511298 A>C	AGO2; TRAPPC9	CHRAC1	.	0.60893
Fam087	DCM	chr2:227503696 G>C	AGFG1	.	.	0.68879
Fam108	DCM	chr19:44008139 A>T		PHLDB3	.	0.74361
Fam126	DCM	chr6:144216524 C>CA	UTRN; STX11; SF3B5	.	.	NA
Fam306	LVNC	chr6:150196298 G>A	PPP1R14C	.	.	0.84031
Fam313	DCM	chr6:26189301 G>GTA	.	H4C4	H1-2; H1-4; H1-6; H2AC11; H2AC12; H2AC4; H2AC8; H2BC10; H2BC11; H2BC12; H2BC5; H2BC9; H3C2; H3C6; H3C7; H3C8; H4C3; H4C7; H4C8; H4C9	NA
Fam334	DCM	chr17:75784788 T>C	UNC13D; WBP2; SAP30BP; TRIM65	H3-3B; MIR4738; UNK	TRIM56; TMEM94	0.84602
Fam338	LVNC	chr15:40953466 C>T	.	CHAC1	DLL4	0.61755
Fam468	HCM	chr1:116485382 A>T	ATP1A1	.	.	0.8964
Fam499	HCM	chr20:44116250 A>G	JPH2	.	.	0.88391
Fam499	HCM	chr5:140711461 A>G	.	VTRNA1-1	.	0.99137
Fam509	HCM	chr19:18557700 C>T	ELL	KXD1	.	0.9407
Fam539	HCM	chr10:68406789 C>T	.	RUFY2	.	0.73967
Fam571	DCM	chr2:189661420 AAAAG>A	.	ASNSD1; ASDURF	WDR17	NA
Fam599	HCM	chr20:298489 C>G	SOX12	ZCCHC3	SOX12	0.94203
Fam687	LVNC	chr4:18021878 A>G	DCAF16	LCORL	LAP3	0.93949
Fam733	HCM	chr9:131270697 A>G	.	FAM78A	.	0.8224
Fam737	HCM	chr2:96816570 G>C	.	CNNM3	.	0.52844
Fam791	ARVC	chr11:67317729 C>T	GRK2; RHOD	RAD9A	.	0.76606
Fam992	ARVC	chr8:15540211 C>A	TUSC3	.	.	0.8979
