# Supplementary material for: De novo and inherited variants in coding and regulatory regions in genetic cardiomyopathies
Source: Hum Genomics. 2022 Nov 10;16:55. doi: 10.1186/s40246-022-00420-0 (PMC9647983; doi:10.1186/s40246-022-00420-0)
Supplement: Supplementary file 2 — Additional file 2: Table S2: All gene targets of noncoding DNVs directly or indirectly associated with cardiomyopathy. [file 40246_2022_420_MOESM2_ESM.pdf]

Table S2: All gene targets of noncoding DNVs directly or indirectly associated with cardiomyopathy. The score is an indication of the strength of the connection between the gene and the disease. The average disease-causing likelihood column reflects the principle that a variant in a gene with high mutation intolerance is more likely to be disease causing.

Gene	Description	Relationship	Score	Average disease-causing likelihood
JPH2	Junctophilin 2	Direct	36.89	39.33%
UTRN	Utrophin	Direct	5.32	55.78%
H1-2	H1.2 Linker Histone, Cluster Member	Indirect	5.04	20.85%
RHOD	Ras Homolog Family Member D	Direct	3.48	45.89%
SAP30BP	SAP30 Binding Protein	Indirect	3.48	0.00%
H3C6	H3 Clustered Histone 6	Indirect	3.32	81.77%
H3-3B	H3.3 Histone B	Indirect	3.25	34.95%
WBP2	WW Domain Binding Protein 2	Indirect	3.06	68.19%
H3C2	H3 Clustered Histone 2	Indirect	3.03	88.22%
H3C7	H3 Clustered Histone 7	Indirect	3.03	81.24%
H3C8	H3 Clustered Histone 8	Indirect	3.03	85.98%
TRIM65	Tripartite Motif Containing 65	Indirect	2.75	0.00%
RUFY2	RUN And FYVE Domain Containing 2	Indirect	2.62	69.67%
H2AC11	H2A Clustered Histone 11	Indirect	2.59	82.88%
H2AC12	H2A Clustered Histone 12	Indirect	2.59	54.14%
H1-6	H1.6 Linker Histone, Cluster Member	Indirect	2.5	15.11%
DLL4	Delta Like Canonical Notch Ligand 4	Indirect	2.39	82.27%
SOX12	SRY-Box Transcription Factor 12	Indirect	2.11	0.00%
VTRNA1-1	Vault RNA 1-1	Indirect	1.91	0.00%
ELL	Elongation Factor For RNA Polymerase II	Indirect	1.85	60.67%
H2AC4	H2A Clustered Histone 4	Indirect	1.57	74.78%
CNNM3	Cyclin And CBS Domain Divalent Metal Cation Transport Mediator 3	Indirect	1.55	65.45%
MIR4738	MicroRNA 4738	Indirect	1.55	0.00%
H2BC9	H2B Clustered Histone 9	Indirect	1.51	83.92%
H2BC11	H2B Clustered Histone 11	Indirect	1.49	76.73%
H2BC12	H2B Clustered Histone 12	Indirect	1.49	69.36%
H2BC5	H2B Clustered Histone 5	Indirect	1.49	68.98%
UNK	Unk Zinc Finger	Indirect	1.46	60.17%
H4C7	H4 Clustered Histone 7	Indirect	1.45	46.88%
H2BC10	H2B Clustered Histone 10	Indirect	1.44	76.56%
CHAC1	ChaC Glutathione Specific Gamma-Glutamylcyclotransferase 1	Indirect	1.39	34.03%
GRK2	G Protein-Coupled Receptor Kinase 2	Direct	1.35	87.88%
ATP1A1	ATPase Na+/K+ Transporting Subunit Alpha 1	Direct	1.17	85.82%
FAM78A	Family With Sequence Similarity 78 Member A	Indirect	1.11	75.79%
PPP1R14C	Protein Phosphatase 1 Regulatory Inhibitor Subunit 14C	Indirect	1.05	62.46%
H4C3	H4 Clustered Histone 3	Direct	0.94	85.81%
H4C4	H4 Clustered Histone 4	Direct	0.94	76.99%
H4C8	H4 Clustered Histone 8	Direct	0.94	89.08%
H4C9	H4 Clustered Histone 9	Direct	0.94	79.34%
TUSC3	Tumor Suppressor Candidate 3	Indirect	0.94	86.57%
SF3B5	Splicing Factor 3b Subunit 5	Indirect	0.71	74.53%
WDR17	WD Repeat Domain 17	Indirect	0.69	24.37%
CHRAC1	Chromatin Accessibility Complex Subunit 1	Indirect	0.6	42.77%
PHLDB3	Pleckstrin Homology Like Domain Family B Member 3	Indirect	0.58	15.67%
TRAPPC9	Trafficking Protein Particle Complex Subunit 9	Direct	0.54	52.47%
ASNSD1	Asparagine Synthetase Domain Containing 1	Indirect	0.43	69.93%
ZCCHC3	Zinc Finger CCHC-Type Containing 3	Indirect	0.43	82.46%
H1-4	H1.4 Linker Histone, Cluster Member	Direct	0.35	70.55%
AGO2	Argonaute RISC Catalytic Component 2	Direct	0.34	97.12%
LAP3	Leucine Aminopeptidase 3	Direct	0.34	57.83%
DCAF16	DDB1 And CUL4 Associated Factor 16	Indirect	0.32	46.35%
TMEM94	Transmembrane Protein 94	Indirect	0.31	77.65%
LCORL	Ligand Dependent Nuclear Receptor Corepressor Like	Indirect	0.3	0.00%
AGFG1	ArfGAP With FG Repeats 1	Direct	0.25	74.75%
H2AC8	H2A Clustered Histone 8	Direct	0.25	86.55%
KXD1	KxDL Motif Containing 1	Direct	0.25	23.74%
RAD9A	RAD9 Checkpoint Clamp Component A	Direct	0.25	42.70%
STX11	Syntaxin 11	Direct	0.25	38.66%
TRIM56	Tripartite Motif Containing 56	Direct	0.25	70.65%
UNC13D	Unc-13 Homolog D	Direct	0.25	29.74%
ASDURF	ASNSD1 Upstream Open Reading Frame	Indirect	0.07	0.00%
