# Supplementary material for: De novo and inherited variants in coding and regulatory regions in genetic cardiomyopathies
Source: Hum Genomics. 2022 Nov 10;16:55. doi: 10.1186/s40246-022-00420-0 (PMC9647983; doi:10.1186/s40246-022-00420-0)
Supplement: Supplementary file 3 — Additional file 3: Table S1: Activity-by-Contact (ABC) model predictions of known cardiomyopathy genes in ventricular cardiomyocytes. [file 40246_2022_420_MOESM3_ESM.pdf]

Table S1: Activity-by-Contact (ABC) model predictions of known cardiomyopathy genes in ventricular cardiomyocytes

Chr	Start	End	Class	Activity base	Target gene	Target gene TSS	Target gene promoter activity quantile	Distance	Is self promoter	Powerlaw contact	Powerlaw contact reference	HiC contact	HiC contact pl scaled	HiC pseudocount	HiC contact pl scaled adj	ABC score numerator	ABC score	Powerlaw score numerator	Powerlaw score	Cell type	ABC score rank
chr1	156106010	156106611	intergenic	13.310302	LMNA	156114669	0.673354	8359	FALSE	0.078941	0.078941	0.060126	0.060126	0.001229	0.061355	0.816651	0.106916	1.050727	0.071948	Ventricular_CMs	1
chr1	201377601	201378015	promoter	19.775764	TNNT2	201377700	0.930789	107.5	TRUE	0.123436	0.123436	0.03435	0.03435	0.001229	0.03558	0.703614	0.123237	2.441042	0.179914	Ventricular_CMs	1
chr10	110636986	110637185	intergenic	16.900542	RBM20	110644396	0.764828	7311	FALSE	0.088697	0.088697	0.022325	0.022325	0.001229	0.023554	0.398079	0.092421	1.499031	0.124352	Ventricular_CMs	1
chr10	119651114	119651512	promoter	18.774625	BAG3	119651369	0.924556	56.5	TRUE	0.123436	0.123436	0.039034	0.039034	0.001229	0.040264	0.755935	0.197683	2.317466	0.287607	Ventricular_CMs	1
chr11	47351831	47352030	genic	17.724121	MYBPC3	47352702	0.887649	772	FALSE	0.123436	0.123436	0.046617	0.046617	0.001229	0.047847	0.84804	0.149349	2.187796	0.186447	Ventricular_CMs	1
chr14	23438169	23438368	intergenic	33.205948	MYH7	23435686	0.920197	2582	FALSE	0.123436	0.123436	0.027188	0.027188	0.001229	0.028417	0.943608	0.117895	4.098811	0.202785	Ventricular_CMs	1
chr2	178807324	178807586	promoter	12.275114	TTN	178807423	0.858604	31.5	TRUE	0.123436	0.123436	0.02878	0.02878	0.001229	0.03001	0.368372	0.14719	1.515192	0.215205	Ventricular_CMs	1
chr2	219419848	219420047	genic	8.815552	DES	219418376	0.799169	1571	FALSE	0.123436	0.123436	0.044384	0.044384	0.001229	0.045613	0.402106	0.057051	1.088157	0.071798	Ventricular_CMs	1
chr3	38645770	38645969	genic	7.954766	SCN5A	38649672	0.747495	3803	FALSE	0.123436	0.123436	0.038343	0.038343	0.001229	0.039572	0.314787	0.074426	0.981905	0.119207	Ventricular_CMs	1
chr3	52452672	52453127	genic	33.339543	TNNC1	52454041	0.804485	1142	FALSE	0.123436	0.123436	0.047516	0.047516	0.001229	0.048745	1.625129	0.219908	4.115302	0.284333	Ventricular_CMs	1
chr6	7541333	7542604	promoter	15.27033	DSP	7541574	0.812979	394	TRUE	0.123436	0.123436	0.032826	0.032826	0.001229	0.034055	0.520034	0.187122	1.884909	0.32017	Ventricular_CMs	1
chr6	118537644	118537843	genic	7.678519	PLN	118548262	0.551552	10519	FALSE	0.064635	0.064635	0.010951	0.010951	0.001229	0.012181	0.093529	0.061553	0.496301	0.120409	Ventricular_CMs	1
chr7	128831493	128831692	genic	15.099412	FLNC	128830428	0.772446	1164	FALSE	0.123436	0.123436	0.049597	0.049597	0.001229	0.050826	0.767444	0.143531	1.863812	0.156495	Ventricular_CMs	1
